# Association of high carbohydrate versus high fat diet with glycated hemoglobin in high calorie consuming type 2 diabetics

**DOI:** 10.1186/2251-6581-12-27

**Published:** 2013-06-14

**Authors:** Zhaleh Shadman, Mohsen Khoshniat, Nooshin Poorsoltan, Mahdieh Akhoundan, Maryam Omidvar, Bagher Larijani, Saeed Hoseini

**Affiliations:** 1 Endocrinology and Metabolism Research Center, Endocrinology and Metabolism Research Institute, Tehran University of Medical Sciences, Tehran, Iran; 2 Diabetes Research Center, Endocrinology and Metabolism Clinical Sciences Institute, Tehran University of Medical Sciences, Tehran, Iran

**Keywords:** Diabetes, Dietary saturated fatty acid, Carbohydrate, Fat

## Abstract

**Background:**

Since both dietary carbohydrate and fatty acids separately affect carbohydrate metabolism, how dietary macronutrients distribution may have different effects on carbohydrate metabolism pathways and regulation of blood glucose especially in diabetic patients.

**Methods:**

In this cross-sectional study 750 type 2 diabetic patients (261 men and 489 women, aged 35–65 years),who at least two years were followed in Diabetes and Metabolic disease Clinic of Tehran University of Medical Sciences, were recruited according to inclusion and exclusion criteria by simple sampling. Dietary data were collected by a validated food frequency questionnaire. Other variables were anthropometric measurements, Stress, physical activity level, Biochemical analyses including fasting and postprandial plasma glucose, Glycated hemoglobin, total cholesterol, low and high density lipoproteins, triglycerides and 25-hydoxy D_3_. Linear regression models were used to assess the association of covariates with the mean concentrations of HbA_1C_ in quintiles and multivariate linear regression model was used to distinguish the impacts of dietary macronutrient composition of the diet.

**Results:**

Carbohydrate and dietary fiber intakes were inversely (*P*: < 0.0001 and 0.003 respectively) and dietary amount and proportion of saturated, mono-unsaturated and poly-unsaturated fatty were positively (*P*: < 0.0001, 0.03, 0.01 and 0.01 respectively) associated with HbA_1C_ concentrations.

Multivariate linear regression macronutrient density model that controlled for age, sex, diabetes duration and calorie intake showed that carbohydrate was inversely associated with HbA_1C_ (*P* < 0.0001, R^2^ = 15%). Results were also the same in the other three models adjusted for stress and exercise levels in model 2, waist circumference and sum of meals in model 3 and serum triglyceride and 25-hydroxy vitamin D in model 4(*P* < .0001, <.0001 and 0.0003 respectively). Calorie intake of 25 Kcal/body weight was identified as a cut of point of the negative effect of dietary carbohydrate and 30 for the positive effect of fat on HbA1c respectively (*P* = 0.04 and 0.03). Moreover, carbohydrate intake was positively (*β* = 0.08, *P* = 0.01) and protein (*β* = −0.04, *P* < 0.0001), SAFA (*β* = −0.04, *P* < 0.0001) and MUFA (*β* = −0.02, 0.07) proportion were negatively associated with increment in calorie intake.

**Conclusion:**

This study showed that the substitution of fat for carbohydrate is associated with low concentrations of HbA1c in high calorie consuming type 2 diabetic patients.

## Background

Medical nutrition therapy is an integral component of diabetes management [1]. In the process of designing an individualized diet, after estimating energy requirement, determining the distribution of dietary macronutrients (percent of carbohydrate, fat and protein of total calorie) is the next step. The current American Diabetes Association (ADA) recommendations suggest a range of carbohydrate intake of between 45% and 65% of total calories, protein 10–20%, total fat ≤ 30%, saturated fatty acids (SFAs) <7%, mono-unsaturated fatty acids (MUFAs) up to 20% and poly unsaturated fatty acids (PUFAs) up to 10% of total calorie [2].

In diabetes management, carbohydrate modification is the first step recommendation and the more emphasis is on it [3], but each macronutrient may involve in carbohydrate metabolism by different biochemical pathways. Since dietary fatty acids (FAs) play a key role in the cell membrane and insulin sensitivity, some fatty acids may induce development of insulin resistance and consequently affect diabetes metabolic control. Observational studies assessing serum or tissues fatty acid composition suggest that insulin resistance is associated with relatively high intakes of saturated fat (e.g. palmitic acid) and low intakes of polyunsaturated fat (e.g. linoleic acid), findings that are supported by recent clinical data [4].

It has been emphasized the ability of low carbohydrate diets to improve glycemic control, hemoglobin A1C (HbA1c) and to reduce medication [5]. In a 2-year follow-up study, HbA1c levels were significantly improved in the carbohydrate restricted diet [6]. A number of short duration trials have demonstrated improvements in insulin resistance with a high total and mono unsaturated fat diet [7–10]; whereas, some others have shown high carbohydrate diets are associated with a better glycemic control [11–13]. In a study, the magnitude of blood glucose decrements was similar after consuming two low-caloric diets (high-glycemic index and the high-fat/low-carbohydrate diets) [14]. Also, several studies have examined the effects of dietary macronutrients on postprandial glucose [15–19] not glycated hemoglobin as a diabetes control indicator.

In several studies so far have been done, the association of dietary macronutrients and calorie intake with the risk of diabetes has been explored. For example, regarding to the type 2 diabetes, high caloric diets were associated to increase [5], high carbohydrate, in some cases, diet was associated to increase [6–8] and in others, associated to decrease [9–12], and diets high in glycemic index and glycemic load were associated to increase [6, 13–16] the risk.

So the question is whether only carbohydrate containing food groups should be taken into diabetes management programs or the amount and type of dietary fat and oil also should be considered. Furthermore, the extent of recommended ranges of macronutrients such as carbohydrate may cause extreme values show opposite effects. Moreover, considering differences in genetic variations, dietary patterns, eating habits and etc. among populations, the proportion of macronutrients in calorie intake may have several effects on glucose metabolism [20, 21].

Based on our best knowledge, no study has evaluated the role of dietary macronutrients on glycemic control in Iranian diabetic patients. The aim of this study was to examine the association of dietary composition of macronutrients with HbA1c and blood glucose in type 2 diabetic patients.

## Methods

This cross-sectional study was approved by the Endocrinology and Metabolism Research Center ethic committee (EC-00146).

### Subjects

In this study 750 type 2 diabetic patients (261 men and 489 women, aged 35–65 years),who at least two years have been followed in Diabetes and Metabolic disease Clinic of Tehran University of Medical Sciences, were recruited according to inclusion and exclusion criteria by simple sampling. Inclusion criteria were 35–65 years old, diagnosis of diabetes after 30 years old, and diabetes mellitus for more than 5 years. Exclusion criteria were insulin therapy, myocardial infarction, angina pectina, stroke, acute liver or renal disease during the past year, chronic inflammation, thyroid disease, vegetarianism, alcohol consumption and pregnancy). At the beginning, the protocol and the aim of the study were fully explained to the subjects and written informed consent was obtained each volunteers.

### Dietary data

A validated 168-item food frequency questionnaire (FFQ) [22] was completed by trained dietitians by face-to-face interviews to assess the usual dietary intakes of participants. To estimate portion sizes, a set of 2-dimensional shapes and in some cases 3-dimensional food models was used. Amounts were documented in household units, eg, teaspoons, cups, and ounces. Data were analyzed for total calorie intake, carbohydrate, protein, saturated and unsaturated fatty acids using adjusted N4 software (Nutritionist: version 4.0; Tinuviel Software, Warrington, United Kingdom).

### Anthropometric measurements

Height was measured with a wall-mounted stadiometer to the nearest 0.1 cm. Weight was determined to the nearest 0.1 kg on the same properly calibrated electronic digital scale, without shoes, with minimal clothing, and after voiding. Two measurements were obtained and averaged; with a third measurement taken if the first two differed by 0.1. Body mass index (BMI) was estimated as the ratio of body weight to height squared and expressed as kg/m^2^. Waist circumference was determined by placing a measuring tape in a horizontal plane around the abdomen just above the right iliac crest. Three measurements were made to the nearest 0.1 cm and averaged.

### Physical activity and stress

Physical activity level was assessed by a validated questionnaire in which nine different metabolic equivalent (MET) levels were ranged on a scale from sleep/rest (0.9 METs) to high-intensity physical activities (> 6 METs) [23]. Over 24 hours, for each activity level, the MET value was multiplied by the time spent at that particular level. The sum of MET-time at each level and, finally, its average was calculated dividing by 24. Measurement of the three related negative emotional states of depression, anxiety and tension/stress was done by the self report validated Depression Anxiety Stress Scales (DASS-42) [24].

### Biochemical tests

Three ml 12-hour fasting state and three ml postprandial brachial vein blood samples were taken and collected into EDTA containing tubes. Samples were centrifuged at 3000 *g* for 10 minutes and 4°C, and promptly plasma aliquoted into separate tubes that were stored at −75°C until analyzed. One ml was stored as whole blood to A1C measurements. Plasma glucose concentration was measured by fluorometric method according glucose oxidase principle (Glucose determination kit, Parsazmun, Tehran, Iran) through auto-analyzer instrument (Hitachi 902, Roche, Basel, Switzerland). Glycated hemoglobin was determined on whole blood sample by HbA1c Pink Kit and DS5 analyzer. The intra assay coefficient of variation (CV%) for glucose and HbA1c were 1.4% and 3.7%, and the inter assay coefficient of variation were 1.9% and 3.5% respectively. Serum triglyceride (TG), total cholesterol (TC), LDL (low density lipoprotein) and HDL (high density lipoprotein) cholesterol were measured by the related biochemical kits (Parsazmun, Tehran, Iran) by the auto-analyzer (Hitachi 902, Roche, Basel, Switzerland). The intra assay CV% were 4.1, 1.3, 2.0 and 1.8 and the inter assay CV% were 4.5, 2.0, 2.3, and 2.0 respectively. Serum 25-hydroxy vitamin D was measured by Enzyme-linked immunosorbent assay (ELAISA) (IDS, Boldon, UK). The intra assay CV% was 5.4% and inter assay CV% 5.5%.

### Statistical analysis

All statistical analyses were performed by SPSS software (version 16.0; SPSS Inc, Chicago) and a p-value < 0.05 showed statistical significance. One sample *T* test was used to compare participants’ dietary intake and quintiles with common nutritional recommendations. In each case that recommendation was as a range, the mean value was used to comparison with each quintile value. First, linear regression models adjusted for age, sex and diabetes duration were used to assess the association of covariates with the mean concentrations of HbA_1C_ in quintiles. In the next step, multivariate linear regression model including the percentage of energy intake from carbohydrate and protein was used to distinguish the impacts of macronutrient composition of the diet and energy intake per body weight and some other variables that may affect diabetes control. Because protein intake is often stable, these models can show the effect of submitting dietary carbohydrate for fat while calorie intake and other covariates are constant.

To determine foods which are responsible for changing in HbA_1C_; fats, oils and other fatty acid containing foods that have correlation with HbA_1C_ (based on Pearson correlation coefficient) were entered into the linear regression model. Pearson correlation was used for assessing the association between dietary macronutrients and fiber. Then to find the true effect of carbohydrate on HbA1c, fiber was added to the model. Since total calorie intake is one of the most effective factors on glucose level control [2, 25], we compared the relation (regression coefficient) of macronutrients and HbA1c between 2 groups of daily calorie intake.

To identify that by increasing of calorie intake in studied population the proportion of which macronutrient is increased, analysis of regression was used for each macronutrient.

## Results

Table 1 shows the basic characteristic of patients. This data show that the mean BMI and glucose control are higher than normal.

**Table 1:** **Basic characteristics of patients**

Variables	Mean ± SD
Age (year)	55 ± 10
BMI (kg/m^2^)	28.8 ± 4.9
Duration of diabetes (month)	125 ± 94
Glycated hemoglobin (%)	8.04 ± 1.93
Serum triglyceride (mg/dl)	165 ± 109
Serum total cholesterol (mg/dl)	166 ± 42
LDL-cholesterol (mg/dl)	89 ± 26
HDL-cholesterol (mg/dl)	50 ± 41

Participants’ dietary intakes in quintiles and its comparison with common nutritional recommendations have been shown in Table 2. In each case that recommendation was as a range the mean recommended range was used to comparison with each quintile value and in the cases that the recommendation was not as a range, the specified value was used. These data show high consumption of calorie in 80%, total and saturated fat in 60% and 80%, and low consumption of fiber in 60% of the participants (Table 2).

**Table 2:** **Comparison of dietary components by quintiles with common nutritional recommendation**

	1	2	3	4	5	Recommendation^1^
Kcal/kg	17.81 (0.42)	24.13 (0.15) ^2^	29.05 (0.19)	35.74 (0.24)	50.85 (1.29)	20–25
% Pro	11.51 (0.14)	13.81 (0.04)	15.08 (0.03)^2^	16.29 (0.04)	18.54 (0.17)	10–20
% carbohydrate	47.41 (0.41)	53.78 (0.12)	57.70 (0.11) ^2^	61.22 (0.13)	67.16 (0.37)	45–65
% total fat	22.92 (0.27)	27.14 (0.09)	30.47 (0.12)^2^	34.35 (0.11)	40.55 (0.49)	<30
% SAFA	6.24 (0.09)	7.90 (0.03)^2^	8.89 (0.03)	9.96 (0.03)	12.73 (0.27)	<7
% MUFA	7.57 (0.11)	9.40 (0.04)	10.69 (0.04)	12.38 (0.06)	15.32 (0.20)^3^	15–20
% PUFA	4.18 (0.07)	5.31 (0.02)	6.15 (0.03)	7.64 (0.06)	10.17 (0.17)	<10
Fiber/Kcal	9.16 (0.17)	11.52 (0.04)	12.98 (0.04)^3^	14.53 (0.06)	18.53 (0.29)	14

^1^In each case that recommendation is as a range the mean recommended range was used to comparison in each quintile.

^2^The mean of variable in this quintile and upper quintiles are more than recommended values.

^3^The mean of variable in this quintile and lower quintiles are less than recommended values.

Age, sex and duration of diabetes adjusted estimates of the mean concentration of HbA_1C_ within the quintiles of stress, physical activity level and dietary variables showed that carbohydrate and dietary fiber intakes were inversely (*P* < 0.0001 and 0.003 respectively) and dietary amount and type of fat were positively (*P*:<0.0001, 0.03, 0.01 and 0.01 for the percentages of total fat, SAFA, MUFA and PUFA from calorie respectively) associated with HbA_1C_ concentrations (Table 3).

**Table 3:** **Sex-age and diabetes duration adjusted estimates of glycated hemoglobin (Hb A1C) with variable quintiles (mean ± SE)**

	1	2	3	4	5	***P*** value^1^	***B*** ^***2***^
Stress score^3^	61.92 (0.61)	76.94 (0.44)	90.65 (0.43)	107.12 (0.60)	134.42 (1.26)		
HbA1c%	7.98 (0.22)	7.66 (0.19)	8.07 (0.20)	8.05 (0.19)	8.46 (0.23)	0.05	0.13
PAL (MET)^4^	1.10 (0.01)	1.34 (0.00)	1.45 (0.00)	1.54 (0.00)	1.77 (0.02)		
HbA1c%	8.28 (0.29)	8.09 (0.19)	8.10 (0.20)	7.90 (0.19)	7.86 (0.22)	0.32	−0.07
Wc (cm)^5^	75.74 (1.88)	89.29 (0.23)	96.22 (0.21)	102.51 (0.21)	113.61 (0.95)		
HbA1c%	8.07 (0.23)	7.67 (0.22)	8.53 (0.25)	7.98 (0.23)	8.08 (0.18)	0.62	0.037
Kcal/kg^6^	17.81 (0.42)	24.13 (0.15)	29.05 (0.19)	35.74 (0.24)	50.85 (1.29)		
HbA1c%	8.27 (0.23)	7.79 (0.19)	7.48 (0.16)	8.23 (0.20)	8.55 (0.28)	0.08	0.12
Protein (g)	51.18 (0.76)	66.42 (0.36)	76.53 (0.35)	90.19 (0.56)	162.21 (37.35)		
HbA1c%	8.17 (0.23)	7.79 (0.17)	7.95 (0.19)	8.09 (0.19)	8.23 (0.24)	0.16	0.09
Pro%^7^	11.51 (0.14)	13.81 (0.04)	15.08 (0.03)	16.29 (0.04)	18.54 (0.17)		
HbA1c%	8.16 (0.23)	8.23 (0.22)	8.34 (0.22)	7.75 (0.19)	7.90 (0.22)	0.15	−0.09
Carbohydrate (g)	200.54 (3.63)	269.87 (1.53)	321.16 (1.60)	381.57 (2.28)	554.27 (14.59)		
HbA1c%	8.02 (0.21)	8.17 (0.19)	7.69 (0.20)	8.31 (0.23)	8.03 (0.19)	0.51	0.04
% carbohydrate^7^	47.41 (0.41)	53.78 (0.12)	57.70 (0.11)	61.22 (0.13)	67.16 (0.37)		
HbA1c%	8.59 (0.27)	8.38 (0.23)	8.11 (0.21)	7.59 (0.17)	7.70 (0.16)	<0.0001	−0.25
Total fat (g)	45.20 (0.67)	57.50 (0.32)	70.87 (0.50)	86.71 (0.64)	135.65 (6.57)		
HbA1c%	7.91 (0.20)	7.79 (0.18)	7.80 (0.17)	8.21 (0.20)	8.51 (0.26)	0.006	0.18
% total fat^7^	22.92 (0.27)	27.14 (0.09)	30.47 (0.12)	34.35 (0.11)	40.55 (0.49)		
HbA1c%	7.66 (0.16)	7.65 (0.17)	8.10 (0.22)	8.06 (0.20)	8.89 (0.28)	<0.0001	0.26
SAFA (g)^8^	11.23 (0.22)	15.11(0.09)	18.22 (0.09)	22.38 (0.14)	35.56 (3.07)		
HbA1c%	7.92 (0.22)	7.86 (0.18)	8.03 (0.18)	7.86 (0.16)	8.55 (0.27)	0.24	0.15
% SAFA^7^	6.24 (0.09)	7.90 (0.03)	8.89 (0.03)	9.96 (0.03)	12.73 (0.27)		
HbA1c%	7.63 (0.19)	7.84 (0.16)	8.10 (0.21)	8.30 (0.24)	8.50 (0.25)	0.03	0.20
MUFA (g)^9^	12.92 (0.21)	17.35 (0.12)	21.74 (0.13)	27.04 (0.20)	42.30 (1.52)		
HbA1c%	7.87 (0.18)	7.85 (0.20)	8.07 (0.18)	7.90 (0.20)	8.53 (0.24)	0.019	0.15
% MUFA^7^	7.57 (0.11)	9.40 (0.04)	10.69 (0.04)	12.38 (0.06)	15.32 (0.20)		
HbA1c%	8.04 (0.19)	7.53 (0.17)	7.98 (0.21)	8.38 (0.21)	8.44 (0.27)	0.017	0.16
PUFA (g)^10^	10.56 (0.24)	14.12 (0.07)	17.36 (0.12)	23.56 (0.26)	42.03 (2.39)		
HbA1c%	7.89 (0.20)	8.09 (0.19)	7.85 (0.18)	8.19 (0.24)	8.21 (0.21)	0.21	0.08
% PUFA^7^	4.18 (0.07)	5.31 (0.02)	6.15 (0.03)	7.64 (0.06)	10.17 (0.17)		
HbA1c%	7.84 (0.20)	8.02 (0.19)	7.76 (0.20)	8.11 (0.20)	8.64 (0.27)	0.014	0.17
Fiber(g)/Kcal^11^	9.16 (0.17)	11.52 (0.04)	12.98 (0.04)	14.53 (0.06)	18.53 (0.29)		
HbA1c%	8.26 (0.21)	8.41 (0.27)	8.02 (0.21)	7.97 (0.16)	7.55 (0.15)	0.003	−0.19

^1^ P-*value* of regression coefficient.

^2^ Regression coefficients.

^3^ Score based on depression anxiety questionnaire.

^4^ Physical activity levels based on each activity related metabolic equivalent.

^5^ Weight circumferences.

^6^ Energy intakes per body weight.

^7^ The daily proportion of this macronutrient from total energy intake.

^8^ Saturated fatty acid.

^9^ Mono-unsaturated fatty acid.

^10^ Poly-unsaturated fatty acid.

^11^ Dietary fiber intakes per 1000 Kilocalorie.

Multivariate linear regression macronutrient density model that controlled for age, sex, DD and calorie intake showed that carbohydrate was inversely associated with HbA_1C_ (*P* < 0.0001, R^2^ = 15%). Results were also the same in the other three models adjusted for stress and exercise levels in model 2, waist circumference and sum of meals in model 3 and serum triglyceride and 25-hydroxy vitamin D in model 4(*P* < .0001, <.0001 and 0.0003 respectively) (Table 4). Analysis of regression adjusted for age, sex, and DD showed no association between the source of carbohydrate (e.g. whole and refined grains, legumes, beans, and fruits) and HbA1c.

**Table 4:** **Dietary and metabolic correlates of glycated hemoglobin (Hb A1**_**C**_ **)** ^**1**^

	Model 1	***P*** value	Model 2	***P*** value	Model 3	***P*** value	Model 4	***P*** value
No. of subjects in model	745		743		740		736	
Model R^2^	0.15		0.17		0.22		0.36	
Variables in model ^2^								
Age	0.68 ± 0.26	.009	0.65 ± 0.26	0.01	0.21 ± 0.34	0.52	0.37 ± 0.45	0.41
Sex	−0.01 ± 0.01	.097	−0.01 ± 0.01	0.06	−0.02 ± 0.01	0.04	−0.03 ± 0.02	0.09
Duration of diabetes	0.005 ± 0.001	<.0001	0.005 ± 0.001	<.0001	0.004 ± 0.001	0.001	0.01 ± 0.002	<.0001
Energy intake per body weight	0.02 ± 0.008	0.021	0.02 ± 0.008	0.02	0.01 ± 0.01	0.30	0.01 ± 0.01	0.34
Percentage of energy from carbohydrate	−0.07 ± 0.01	<0.0001	−0.07 ± 0.01	<.0001	−0.08 ± 0.02	<0.0001	−0.07 ± 0.02	0.0003
Percentage of energy from protein	−0.06 ± .04	0.146	−0.07 ± 0.04	0.08	−0.18 ± 0.05	0.001	−0.22 ± 0.06	0.001
Mean stress level	–	–	0.004 ± 0.004	0.37	0.005 ± 0.005	0.32	0.002 ± 0.006	0.68
Mean exercise level	–	–	–0.52 ± 0.42	0.22	–0.70 ± 0.52	0.17	–0.30 ± 0.61	0.61
Waist circumference	–	–	–	–	–0.003 ± 0.01	0.81	0.01 ± 0.01	0.39
Mean sum of meals a day	–	–	–	–	0.08 ± 0.17	0.64	–0.004 ± 0.19	0.98
Serum triglyceride	–	–	–	–	–	–	0.003 ± 0.001	0.01
Serum 25 hydroxy vitamin D	–	–	–	–	–	–	−0.004 ± 0.002	0.11

^1^ Multivariate linear regression macronutrient density models.

^2^ Parameter estimate.

Pearson correlation showed that dietary carbohydrate was positively (r = 0.78, p < 0.0001) and protein (r = −0.07, p = 0.13) and fat were negatively associated (r = −0.23, p < 0.0001) to dietary fiber. Controlling for fiber in macronutrients density regression model showed a reduction of carbohydrate regression coefficient (P = 0.001, β = −0.087).

Among all of fat containing food items, animal fat, Hydrogenated oils, High fat dairy products, Butter, cream, and Ground meat were positively associated with HbA_1C_ variations (data not shown). In the next step that this determined food items were entered in a regression model showed that consumption of hydrogenated vegetable oils and ground meat were significantly associated with HbA_1C_ (*P* < 0.0001 and *P* = 0.007 respectively)(Table 5).

**Table 5:** **Association of food items with HbA**_**1C**_

	B ± SE	***P*** value
Animal fat	−0.04 ± 0.03	0.23
Hydrogenated oil	0.04 ± 0.007	<0.0001
High fat dairy products	0.02 ±0.01	0.10
Butter and cream	0.007 ± 0.01	0.58
Ground meat	0.09 ± 0.05	0.007

Then we compared the regression coefficients of macronutrients with HbA1c between 2 groups based on calorie intake classification because it was assumed that the effect of macronutrients on blood glucose may be affected by a cut of point of calorie intake.

Table 6 shows that the inverse effect of carbohydrate on HbA1c at the levels of calorie intake lower than 25 kcal/body weight significantly is stronger than higher levels of calorie intake (*P* = 0.04). Also, calorie intake of 30 Kcal/body weight was identified as a cut of point of the positive effect of dietary total fat on HbA1c (*P* = 0.03). In contrast, association of dietary SAFA with HbA1c was stronger at the levels higher than the cut of point of 27 Kcal/Kg (*P* = 0.04). In regard to dietary MUFA, PUFA and fiber no significant differences were identified at any levels of calorie intake (Table 6).

**Table 6:** **Regression coefficients of macronutrients with HbA1c between 2 groups based on calorie intake**

	***Kcal***/***Kg levels of significancy***	Regression coefficients	( ***p***- ***value***)	***p***-***value of differences between 2 coefficients***
% Carbohydrate	25	−0.10 ± 0.03	0.001	0.04
		−0.04 ± 0.01	0.004	
% Total Fat	30	0.09 ± 0.02	0.000	0.03
		0.04 ± 0.02	0.062	
% SAFA	27	.052 ± 0.03	.425	0.04
		.226 ± .058	.000	
% MUFA	–	–	–	–
% PUFA	–	–	–	–
% Fiber	–	–	–	–

Multivariate regression model showed that carbohydrate proportion was positively (*β* = 0.08, *P* = 0.01) and protein (*β* = −0.04, *P* < 0.0001), SAFA (*β* = −0.04, *P* < 0.0001) and MUFA (*β* = −0.02, 0.07) proportion were negatively associated with increment in calorie intake (Table 7).

**Table 7:** **Association of calorie intakes with dietary macronutrients**

	Coefficient ± SE	***p***-***value***
% carbohydrate	0.08 ± .03	0.01
% protein	–0.04 ± 0.01	0.0001
% total fat	–0.01 ± 0.02	0.64
% SAFA	–0.04 ± 0.01	0.0001
% PUFA	0.02 ± 0.009	0.03
% MUFA	–0.02 ± 0.01	0.07

## Discussion

This study showed that in type 2 diabetic patients on oral hypoglycemic agents, the substitution of fat for carbohydrate (ie, diets high in carbohydrate versus low in fat and saturated fat) is associated with low concentrations of HbA1c independent of age, sex, diabetes duration, stress and physical activity level, waist circumference, calorie intake, sum of daily meals, serum triglyceride and 25(OH) calciferol. By inserting the dietary fiber intake in the regression model, the regression coefficient was decreased but still significant.

Noticeable, in comparison to dietary macronutrients distribution recommendation, the intakes of total and saturated fat were high, dietary fiber was low and carbohydrate was in the recommended range. This composition of the diet has been observed in another studies on Iranian diabetics [26], the population based study of Tehran Lipid and Glucose Study [27] and in some other studies [12, 28–31]. Also, results in Table 7 showed that in this studied Iranian patients along with increment in calorie intake, among all dietary macronutrients, proportion of dietary carbohydrate and PUFA increased. In the other words, increment in calorie intake was associated to the intake of foods high in carbohydrate especially grains (e.g. bread and rice) and greasy foods prepared with high PUFA vegetable oils. Furthermore, the negative association between calorie intake and dietary protein and SAFA showed that our patients on high calorie diets, because of personal preferences or limitation in financial ability, did not increase the consumption of high protein containing foods (e.g. meat and dairy products) and foods high in SAFA.

In a population-based study on non-diabetic persons, total dietary fat and saturated fat were positively associated with HbA1c; but the association of PUFA and MUFA was not statistically significant [32]. Several studies have indicated beneficial effect of high MUFA diets, for example Mediterranean diet in prevention and managing diabetes [9, 33–35]. One meta-analysis including long-term trials with duration of at least 6 months comparing high-MUFA (>12% of total energy content) versus low-MUFA (≤12% of total energy content) diets on glycemic control in participants with abnormal glucose metabolism found that high MUFA diets appear to be effective in reducing HbA1c [36]. Energy restriction was applied in seven of nine included trials.

In a study on overweight subjects with relatively high serum insulin, low carbohydrate and low fat hypocaloric diets both made a reduction in serum glucose but the reduction was not statistically significant. However, the low carbohydrate diet led to an improvement in insulin sensitivity [37]. These results were constant on diabetics; so there was a trend toward a greater decrease in mean fasting glucose level and glycosylated hemoglobin values and an improvement in insulin sensitivity of diabetic subjects on the hypocaloric low-carbohydrate diet, as compared with those on the low-fat diet [38]. It should be noted that the participants’ diet in these two studies was associated with reduction in calorie intake. However, the energy intake in our study was changeless during the past year and was higher than recommended values. It seems that the beneficial effects of low carbohydrate and low fat diets in these two studies are attributable to the calorie restriction. Such an effect was not involved in our study.

In contrast to the commonly held view, this study showed that type 2 diabetic patients on high carbohydrate and low saturated fat diet have a better blood glucose control. Our results is according to the conclusion of two meta-analysis of the evidence that has shown high carbohydrate, high fiber diets compared to moderate carbohydrate, low fiber diets are associated with lower values for fasting, postprandial and average plasma glucose; hemoglobin A_1c_[39, 40]. This effect may be partly explained by carbohydrate and lipid metabolism pathways. Carbohydrate as the easiest to break down is the body proffered energy source. Carbohydrate effect in stimulating insulin secretion leads to increase in carbohydrate, but a decrease in fat oxidation [41]. So, it can be expressed that fat oxidation is determined primarily by the gap between total energy expenditure and the amount of energy ingested in the form of carbohydrate and protein, rather than by the amount of fat consumed [42]. Indeed, it seems that the effect of dietary macronutrient composition on several aspects of metabolic control may be the most important in a high calorie diet compared to low calorie or iso-caloric diet; because in low calorie or iso-caloric diet all of ingested and absorbed macronutrients should be oxidized to supply body needs. But, if the calorie intake is more than energy expenditure, more dietary fat may remain and induce weight gain, change cell membrane fatty acid composition and increase insulin resistance [33]. Also, it has been determined that saturated fatty acid oxidation rate is slower than unsaturated [43]. In the other word, dietary saturated fat has more opportunities to enter cell membrane, affect membrane fluidity, and promote insulin resistance.

In our study, the reason of no significant relationship between energy intake and HbA1c might be due to the increment of carbohydrate proportion of the diet following to increment in caloric intake, that high carbohydrate may attenuate the effects of high calorie intake on blood glucose control.

In addition, analysis of data showed that calorie intakes of 25 and 30 kcal/kg body weight were respectively the cut off points of the effects of carbohydrate and total fat on HbA1c; so, the association coefficients of dietary carbohydrate or fat with HbA1c were significantly higher in the lower values. In respect to dietary saturated fat, this association is more pronounced at higher calorie intake levels with cut off point of 27 kcal/kg body weight.

When caloric intake exceeds 27 kcal/kg body weight, dietary saturated fatty acids would probably replace in cells membrane, altering insulin receptors and insulin secretion, so promoting insulin resistance.

Other beneficial effects of high carbohydrate diet in our study may be related to high contents of dietary fiber, Fructo-oligosaccharides, resistant starch and indigestible carbohydrates that may increase peripheral insulin sensitivity and insulin secretion and decrease glucose release of the liver [44–47].

## Conclusions

In this study both BMI and calorie intakes were more than appropriate levels regard to Participants’ characteristics. Furthermore, more total and saturated fat consumption may be responsible for failure to control blood glucose. Also, it appears that diabetic’s diet, which consuming high calorie diets should be high in carbohydrate to fasilate improvement in glycemic control.

## Abbreviations

ADA: American diabetes association
BMI: Body mass index
CV: Coefficient of variation
DASS: Depression anxiety stress scales
EDTA: Ethylenediaminetetraacetic acid
ELAISA: Enzyme-linked immunosorbent assay
FFQ: Food frequency questionnaire
FAs: Fatty acids
HbA1c: Hemoglobin A1C
HDL: High density lipoprotein
LDL: Low density lipoprotein
MET: Metabolic equivalent
MUFAs: Mono-unsaturated fatty acids
PUFAs: Poly unsaturated fatty acids
SFAs: Saturated fatty acids
SPSS: Statistical package for the social sciences
TG: Triglyceride
TC: Total cholesterol.
